# SELF-EFFICACY MEDIATES THE RELATIONSHIP BETWEEN SEXUAL EDUCATION AND PREVENTION OF SEXUALLY-TRANSMITTED DISEASES AWARENESS AMONG SECONDARY SCHOOL STUDENTS IN PADANG CITY, INDONESIA

**DOI:** 10.21010/Ajidv19i1.7

**Published:** 2024-10-25

**Authors:** NINDREA Ricvan Dana, DARMA Ika Yulia

**Affiliations:** 1 Department of Medicine, Faculty of Medicine, Universitas Negeri Padang, Bukittinggi, Indonesia, 26181; 2 Department of Midwifery, Universitas Syedza Saintika, Padang, Indonesia, 25132

**Keywords:** Adolescent, Sexually transmitted diseases, Self-efficacy, Sexual education

## Abstract

**Background:**

Adolescence is a crucial stage for developing behaviors that impact long-term sexual and reproductive health, particularly in LMICs like Indonesia.

This study examined the relationship between sexual education, self-efficacy, and awareness of sexually transmitted diseases (STDs) prevention among secondary school students in Padang City, Indonesia.

**Materials and Methods:**

A cross-sectional study was conducted with 254 students aged 12-15 years, using self-administered questionnaires and a multi-stage stratified cluster sampling technique. Data analysis was performed using Structural Equation Modelling (SEM).

**Results:**

SEM revealed significant relationships between self-efficacy and STDs prevention (β=0.528, P<0.001), self-efficacy and sexual education (β=0.220, P=0.001), and sexual education and STDs prevention (β=0.218, P=0.001).

**Conclusion:**

These findings highlight the importance of self-efficacy as a mediator in enhancing the effectiveness of sexual education programs, suggesting that boosting students’ confidence in their ability to protect themselves can improve STDs prevention behaviors.

## Introduction

Adolescence is a critical developmental stage where individuals begin to form behaviors that significantly impact their long-term health and well-being (Abdul *et al.*, 2018). Among these behaviors, sexual practices and the prevention of sexually transmitted diseases (STDs) are particularly concerning, especially in LMICs like Indonesia, where comprehensive sexual education and access to healthcare resources are often limited (Nindrea *et al.*, 2020).

Reproductive health and STDs prevention during adolescence are crucial as they influence sexual and reproductive behaviors in adulthood. However, awareness and knowledge of STDs prevention remain low among Indonesian adolescents (Addoh *et al.*, 2017). The 2018 Basic Health Survey revealed that only 28.6% of high school students had adequate knowledge of HIV/AIDS prevention, reflecting a broader issue of insufficient education on STDs in the country (MoH of the Republic of Indonesia, 2018). This lack of awareness is alarming, considering that the WHO estimates that more than one million people are infected with STDs daily, with half of these cases occurring among individuals aged 15-24 years (Closson *et al.*, 2018).

Sexual education plays a fundamental role in shaping adolescents’ understanding of reproductive health and STDs prevention (Ibanez *et al.*, 2017). However, the effectiveness of sexual education programs can be significantly influenced by the self-efficacy of the students receiving them. Self-efficacy, defined as an individual’s belief in their ability to execute behaviors necessary to achieve specific goals, is crucial in determining whether adolescents will apply the knowledge gained from sexual education to engage in protective behaviors (Rhodes *et al.*, 2017). Adolescents with high self-efficacy are more likely to use condoms consistently, seek information about sexual health, and avoid risky sexual behaviors. In contrast, those with low self-efficacy may struggle to translate knowledge into action, increasing their vulnerability to STDs (Painter *et al.*, 2019).

Effective sexual education programs not only provide information but also empower students to believe in their ability to protect themselves from STDs (Serier *et al.*, 2021). This empowerment is essential in translating knowledge into preventive behaviors, making self-efficacy a critical factor in the success of sexual education initiatives.

Despite the recognized importance of self-efficacy in health behavior change, there is limited research exploring how self-efficacy mediates the relationship between sexual education and STD prevention awareness among adolescents in Indonesia. Understanding this mediation process is crucial for developing targeted interventions that can effectively enhance STDs prevention among Indonesian youth.

## Materials and Methods

### Study design and sample

We performed a school-based cross-sectional study in Padang City, West Sumatra Province, Indonesia. The study participants were secondary school students in Padang City. The inclusion criteria were: 1) aged 12-15 years; 2) able to read and write in Bahasa Indonesia. We excluded students who were not present at the time of the study. We calculated the sample size based on the assumption that 21% (p = 0.21) of the students had inadequate prevention of STDs, with an arbitrary margin of error of 5%. This resulted in a required sample size of 254 students (Nindrea *et al.*, 2024).

### Sampling Techniques

The sampling technique used in this study was multistage stratified clustered sampling. In the first stage, we obtained the secondary school database from the Padang City Education Office, West Sumatra Province, Indonesia. Two schools were then randomly selected. In each school, we performed a stratified random sampling of classrooms to select two classrooms per grade level using the list of classrooms provided by the school. All students in each sampled classroom were invited to participate in the study.

### Operational definition

For self-efficacy, we asked, “*How confident were you that you could find and use reliable information about STDs and their prevention?*” and “*How confident were you in recognizing the symptoms of sexually transmitted diseases?*” with responses given on a Likert scale. For sexual education, we asked, “*Did you ever attend a program or class on sexual education in school?*” with a yes or no response. Respondents used a likert scale to answer questions assessing their awareness of STDs prevention (Folasayo *et al.*, 2017). The survey instrument underwent validity and reliability testing, achieving a Cronbach’s alpha value greater than 0.7, indicating acceptable internal consistency.

### Data collection technique

The data collectors visited the selected school and provided a letter explaining the study details to the school administrators. Data collectors then arranged a date for data collection, and provided information regarding the study, rights to refuse participation, voluntary nature of participation, as well as privacy and confidentiality. Data collectors then obtained verbal consent from participating students.

The students then filled out the self-administered questionnaire. The data collectors collected the questionnaires, checked them for completion and accuracy, and entered the data from the remaining paper questionnaires into an electronic database, ensuring its validity. The institutional review board (IRBs) approved a waiver for the need for written consent from minors to avoid compromising the anonymity, privacy, and confidentiality of the study participants. We provided secondary school students with information and asked for verbal informed consent.

### Data management

Data from completed paper questionnaire were entered into an electronic database using the KoboToolbox platform with a team of trained data entry staff. Investigators compared the unique ID and time of survey completion according to metadata with the unique ID in the informed consent form for quality control purpose and resolved the discrepancies at the end of each day in order to ensure that all data were complete and provided accurate information of the study participants.

### Data analysis

A descriptive analysis was conducted in this study to assess the characteristics of the respondents. The reliability and validity of the measures, including Cronbach’s alpha, composite reliability (CR), and average variance extracted (AVE), were examined using SmartPLS. Additionally, SEM techniques were employed to test the research hypotheses.

### Ethical considerations

This study received ethical approval from the ethics committee of the Dr. M. Djamil Hospital, Padang (Ref. No. DP.05.04/D.XVI.X/538/2023).

## Results

Characteristics of the study participants are as shown in Table 1.

**Table 1:** Characteristics of respondents

	Characteristic	Frequency (%)
**Ethnicity**		
	Minangnese	146 (57.5)
	Javanese	36 (14.2)
	Bataknese	8 (3.1)
	Sundanese	5 (2.0)
	Others	59 (23.2)
**Father’s occupation**		
	Civil servant/ state enterprise	54 (21.3)
	Private sector employee	93 (36.6)
	Small-scale vendors/ service providers	42 (16.5)
	Business owner/ entrepreneur	26 (10.2)
	Laborer/ manual workers	18 (7.1)
	Agriculture/ fishery	19 (7.5)
	Independent professions (e.g., lawyers, architects)	2 (0.8)
**Father’s education**		
	Junior high school	38 (15.0)
	Senior high school	86 (33.9)
	Vocational certificate	1 (0.4)
	Associate’s degree	71 (28.0)
	Bachelor’s degree	51 (20.1)
	Higher than bachelor’s degree	7 (2.8)
**Mother’s occupation**		
	Housewife	174 (68.5)
	Civil servant/ state enterprise	15 (5.9)
	Private sector employee	22 (8.7)
	Small-scale vendors/ service providers	39 (15.4)
	Business owner/ entrepreneur	4 (1.6)
**Mother’s education**		
	Primary school	2 (0.8)
	Junior high school	45 (17.7)
	Senior high school	102 (40.2)
	Vocational certificate	4 (1.6)
	Associate’s degree	80 (31.5)
	Bachelor’s degree	21 (8.3)
**Household monthly income**		
	< 1,000,000 IDR	0
	1,000,000 to 2,000,000 IDR	31 (12.2)
	2,000,001 to 3,000,000 IDR	61 (24.0)
	3,000,001 to 4,000,000 IDR	96 (37.8)
	4,000,001 to 5,000,000 IDR	47 (18.5)
	5,000,001 to 6,000,000 IDR	19 (7.5)
**Religion**		
	Islam	241 (94.9)
	Christianity	13 (5.1)

Table 1 shows that that the majority of participants were of Minangnese ethnicity who identified as Muslims. Most commonly, participants had fathers who were employed in the private sector and had senior high school education, and mothers who were housewives with senior high school education. Most participants reported 3,000,001 to 4,000,000 IDR of monthly household income.

Results from the confirmatory factor analysis are shown in *Table 2*.

**Table 2:** Results from the confirmatory factor analysis

Constructs/ factors	SFL
*Sexual education (α=0.814; CR=0.824; AVE=0.679 )*	
S - Did you ever attend a program or class on sexual education in school*	0.750
*Self-Efficacy (α=0.825; CR=0.833; AVE=0.681)*	
SE1 - How confident were you that you could find and use reliable information about STDs and their prevention*	0.802
SE2 - How confident were you in recognizing the symptoms of sexually transmitted diseases*	0.790
*Prevention of STDs awareness (α=0.882; CR=0.875; AVE=0.674)*	
PS1 - I do not want to give salaam when greeting someone who is HIV/AIDS positive	0.777
PS2 - Adolescents who are HIV-positive should be banned from attending schools with HIV-negative students	0.788
PS3 - Adolescents who are HIV-positive should not swim in a pool with HIVnegative students	0.811

α = Cronbach’s alpha; AVE, average variance extracted; CR, composite reliability; SFL, standardized factor loadings

Table 2 showed that all SFL were > 0.60 and statistically significant (P<0.05). All the variables studied were found to be reliable (α ≥ 0.70). The research constructs demonstrated satisfactory convergent validity (SFL > 0.70, CR > 0.8, AVE ≥ 0.5).

Results from the discriminant validity are shown in *Table 3*.

**Table 3:** Results from the discriminant validity

	Constructs	A	B	C
A	Sexual education	***0.755***		
B	Self-efficacy	0.335	***0.735***	
C	Prevention of STDs	0.301	0.289	***0.762***

Bold values indicated the AVE, while the remaining values represented the squared interconstruct correlations

Table 3 showed that the AVE of each latent construct needed to exceed the squared correlations between that construct and the other latent constructs. The analysis confirmed that the requirements for discriminant validity were met.

**Figure 1:**
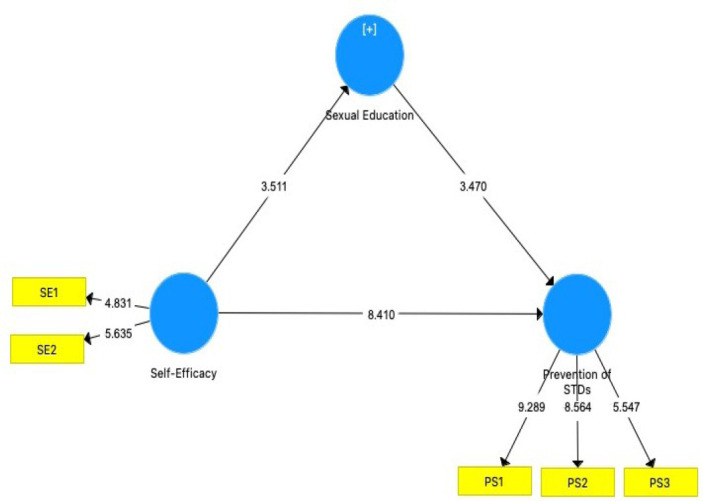
Path analysis diagram

Path analysis is presented in *Table 4 and Figure 1*.

**Table 4:** The results of path analysis

Path specified	Coefficient (β)	t-value	P	Conclusion
Self-efficacy -> Prevention of STDs	0.528	8.410	<0.001	Accepted
Self-efficacy-> Sexual education	0.220	3.511	0.001	Accepted
Sexual education -> Prevention of STDs	0.218	3.470	0.001	Accepted

Table 4 and Figure 1 illustrate that there were relationships between self-efficacy and prevention of STDs (β=0.528, P=<0.001), self-efficacy and sexual education (β=0.220, P=0.001) and sexual education and prevention of STDs (β=0.218, P=0.001).

## Discussion

The results of this study align with and expand upon existing research on the role of self-efficacy in health behavior change, particularly in the context of sexual education and STDs prevention. Previous studies highlighted the role of self-efficacy in determining the success of sexual education programs (Inthavong *et al.*, 2020; Nyasulu *et al.*, 2018). Their research suggested that sexual education is most effective when it not only provides factual information but also includes components that empower students, enhancing their belief in their capacity to engage in safe sexual behaviors. The results of our study confirm this finding, showing that students with higher levels of self-efficacy are more likely to translate sexual education into meaningful awareness and preventive actions regarding STDs.

Moreover, another studies further supports our findings by illustrating how self-efficacy functions as a mediating factor in health behavior interventions (Inthavong *et al.*, 2020; Barral *et al.*, 2020). Their study, conducted in a sample of adolescents in the developing countries, showed that self-efficacy mediated the relationship between sexual education and the adoption of protective sexual behaviors, such as the consistent use of condoms and the avoidance of risky sexual encounters.

The relationship between sexual education and self-efficacy has also been explored in the context of broader reproductive health programs (Wahab *et al.*, 2021). Previous studies found that when adolescents are empowered through educational programs that boost their self-efficacy, they are better equipped to navigate complex sexual health decisions (Nindrea *et al.*, 2021; Fahmi *et al.*, 2021). Their study showed that adolescents who reported higher levels of self-efficacy were more likely to seek out sexual health information, communicate with partners about sexual health, and access healthcare services for STD testing and treatment. These results closely mirror the results of our study, in which self-efficacy was found to significantly mediate the relationship between sexual education and STDs prevention awareness.

In the context of LMICs, a study found that the effectiveness of sexual health programs in Indonesia is often limited by a lack of access to comprehensive education and healthcare resources (Nindrea *et al.*, 2024). Our study expands on their findings by demonstrating that while sexual education is indeed important, it is the students’ self-efficacy that plays a crucial role in determining whether they will act on the information they receive. This highlights the need for sexual education programs in LMICs like Indonesia to focus on empowering students, not just informing them.

Despite the promising insights, this study has several limitations. First, the cross-sectional design limits our ability to infer causality between the variables. Longitudinal studies are recommended to examine how self-efficacy and sexual education influence STDs prevention awareness over time. Second, future study should aim to replicate this study in diverse settings to validate the findings.

## Conclusion

In conclusion, self-efficacy is a vital mediator in the relationship between sexual education and awareness of STDs prevention. The findings highlight the importance of not only delivering knowledge but also empowering adolescents to apply this knowledge effectively in their lives. For sexual health interventions to be successful, especially in settings where comprehensive sexual education is lacking, enhancing adolescents’ self-efficacy should be a key focus. Implementing such programs in schools could have a profound impact on reducing the prevalence of STDs among adolescents, ultimately contributing to better public health outcomes.
